# Muscle Fatigue in the Temporal and Masseter Muscles in Patients with Temporomandibular Dysfunction

**DOI:** 10.1155/2015/269734

**Published:** 2015-03-26

**Authors:** Krzysztof Woźniak, Mariusz Lipski, Damian Lichota, Liliana Szyszka-Sommerfeld

**Affiliations:** ^1^Department of Orthodontics, Pomeranian Medical University of Szczecin, 70111 Szczecin, Poland; ^2^Department of Preclinical Conservative Dentistry and Preclinical Endodontics, Pomeranian Medical University of Szczecin, 70111 Szczecin, Poland; ^3^Department of Conservative Dentistry, Pomeranian Medical University of Szczecin, 70111 Szczecin, Poland

## Abstract

The aim of this study is to evaluate muscle fatigue in the temporal and masseter muscles in patients with temporomandibular dysfunction (TMD). Two hundred volunteers aged 19.3 to 27.8 years (mean 21.50, SD 0.97) participated in this study. Electromyographical (EMG) recordings were performed using a DAB-Bluetooth Instrument (Zebris Medical GmbH, Germany). Muscle fatigue was evaluated on the basis of a maximum effort test. The test was performed during a 10-second maximum isometric contraction (MVC) of the jaws. An analysis of changes in the mean power frequency of the two pairs of temporal and masseter muscles (MPF%) revealed significant differences in the groups of patients with varying degrees of temporomandibular disorders according to Di (*P* < 0.0000). The study showed an increase in the muscle fatigue of the temporal and masseter muscles correlated with the intensity of temporomandibular dysfunction symptoms in patients. The use of surface electromyography in assessing muscle fatigue is an excellent diagnostic tool for identifying patients with temporomandibular dysfunction.

## 1. Introduction

According to various reports, the prevalence of functional disorders in the population aged 3–74 years ranges from 7% to 84% [1–6]. According to Luther, such a large discrepancy is probably the result of using different methodologies in assessing these types of disorders [7].

A review of epidemiological studies conducted by McNeill [1] indicates that about 75% of the population has at least one objective symptom of functional disorders, whereas only 33% reports subjective symptoms. It is also estimated that a need for treatment is expressed by only 5-6% of a large population of people with temporomandibular dysfunction. Only a 7% rate in the occurrence of subjective symptoms of temporomandibular dysfunction was reported by List et al. [8] in a group of 826 children and adolescents aged 12 to 18 years.

Similar conclusions regarding the disparity between the prevalence of subjective symptoms and the recorded evidence of functional disorders were presented by Mohlin et al. [2] following a critical review of 58 studies. A significant difference in the prevalence of subjective and objective symptoms was also revealed by a meta-analysis of 51 studies in the area of temporomandibular dysfunction conducted by de Kanter et al. [9]. The incidence of subjective symptoms was found to range between 6% and 93% and the incidence of objective symptoms, confirmed by clinical examination, between 0% and 93%. A significant discrepancy between the prevalence of subjective symptoms and objective symptoms, which are clinically confirmed signs of temporomandibular dysfunction, was also observed by Suvinen et al. [10]. An analysis of this phenomenon conducted by the authors revealed a relatively weak, on the borderline of statistical significance, correlation between subjective symptoms and objective symptoms observed in routine dental examination. Luther [11] demonstrates beyond any doubt that the disparity connected with a higher incidence of objective symptoms in relation to subjective symptoms is a characteristic feature of temporomandibular dysfunction.

In the light of the evidence presented, expanding the repertoire of modern noninvasive diagnostic methods should result in obtaining more objective research results [12–14].

The aim of this study is to evaluate muscle fatigue in the temporal and masseter muscles in patients with temporomandibular dysfunction.

## 2. Materials and Methods

The research was approved by the Ethics Committee of the Pomeranian Medical University in Szczecin, Poland (number BN-001/45/07) as being consistent with the principles of Good Clinical Practice (GCP). All the patients were informed about the aim and research design and they gave their consent in order to participate.

Two hundred volunteers (100 females and 100 males) aged 19.3 to 27.8 (mean 21.50, SD 0.97) referred to the Orthodontic Department of the Pomeranian Medical University in Szczecin participated in this study. Inclusion criteria were that the participants should be aged between 19 and 28 years and express consent to participate voluntarily in the study. As a result of the application of the adopted exclusion criteria listed in Table 1, 174 of these (93 females and 81 males) qualified for further examination.

Anamnestic interviews which included the patients' general medical history as well as detailed information about their masticatory motor system were conducted. The patients were divided according to a three-point anamnestic index of temporomandibular dysfunction (Ai).

The assessment of the function of the masticatory motor system included clinical as well as electromyographic examinations. The former involved visual and auscultatory assessment as well as palpation and made it possible to qualitatively and quantitatively evaluate the function of the masticatory system. The clinical index of temporomandibular dysfunction was used for the analysis of the data obtained from the clinical study (Table 2). The interpretation of the results of the clinical index of temporomandibular dysfunction (Di), based on the total number of points obtained during the tests, was performed according to the following model (Table 3) [15, 16].

EMG recordings were performed using a DAB-Bluetooth Instrument (Zebris Medical GmbH, Germany). During these recordings each patient was sitting on a comfortable chair without head support and was instructed to assume a natural head position during electromyographic examination.

Surface EMG signals were detected by four silver/silver chloride (Ag/AgCl), disposable, self-adhesive, bipolar electrodes (Naroxon Dual Electrode, Naroxon, USA) with a fixed interelectrode distance of 20 mm. The electrodes were positioned on the anterior temporal muscles and the superficial masseter on both the left and the right sides parallel to the muscular fibres, for the anterior temporal muscle: vertically along the anterior margin of the muscle; for the masseter muscle: parallel to the muscular fibres with the upper pole of the electrode at the intersection between the tragus-commissura labiorum and exocanthion-gonion lines. A reference electrode was placed inferior and posterior to the right ear [17].

Before the recordings, in order to reduce impedance, the skin was carefully cleaned with 70% ethyl alcohol and dried. The EMG procedures were performed 5 minutes later.

The DAB-Bluetooth Instrument was interfaced with a computer which presented the data graphically and recorded it for further analysis. The EMG signals were amplified, digitized, and digitally filtered.

Muscle fatigue was evaluated on the basis of a maximum effort test. The test was performed during a 10-second maximum isometric contraction (MVC) of the jaws. Analysis of the mean power frequency (MPF%), as a variable independent of the complex impedance of the measurement system, did not require the use of a normalization process.

The asymmetry between the activity of the left and the right jaw muscles was quantified by the Asymmetry Index (As). It ranges from 0% (total symmetry) to 100% (total asymmetry) [18–20]:

(1) $$\begin{array}{c} \text{As}=\frac{\sum_{i=1}^{N}\left|R_{i}-L_{i}\right|}{\sum_{i=1}^{N}\left(R_{i}+L_{i}\right)}\cdot 100. \end{array}$$

The Kruskal-Wallis test, the median, and the Mann-Whitney *U* test were used to verify the hypotheses relating to the existence or absence of differences between the mean values of the independent variables. The statistical significance for verifying all the hypotheses was set at *P* = 0.05.

## 3. Results

**Figure 1:**
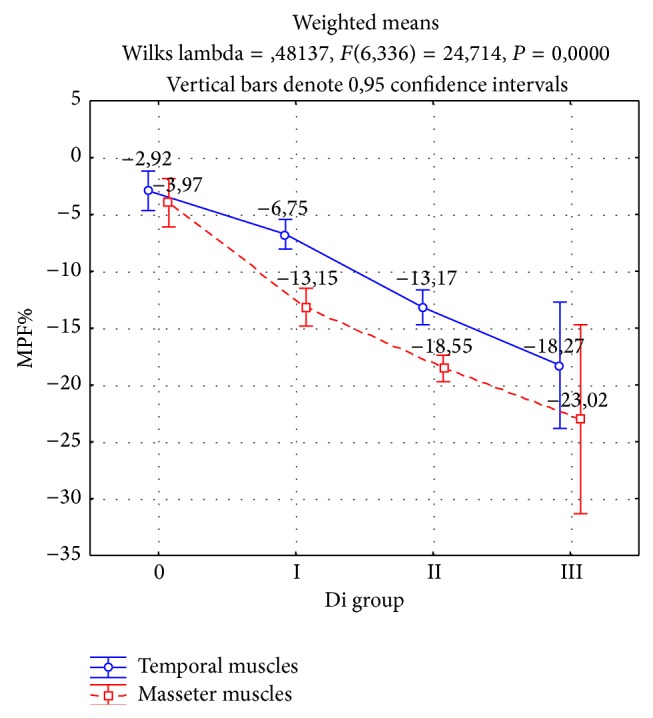
Changes in mean power frequency (MPF%) of muscles during 10 s of maximal voluntary contraction in intercuspal position depending on the temporomandibular dysfunction.

The analysis of changes in the mean power frequency of the two pairs of temporal and masseter muscles (MPF%) showed significant differences in the groups with varying severities of temporomandibular dysfunction according to the Di index (*P* < 0.0000, Table 4, Figure 1). There was a significant tendency to increased fatigue in the tested muscles in direct proportion to the severity of the temporomandibular dysfunction according to the Di.

Resistance to muscle fatigue was modified at the level of the type of muscles examined (*P* < 0.0000). There was always greater depletion of the interference signal in the case of the masseter muscles relative to the temporal muscles.

Changes in the mean power frequency (MPF%) of temporal muscles during 10 s of maximum voluntary contraction were the lowest in the group with no symptoms of temporomandibular dysfunction (−2.92%). Significantly higher depletion of the interference signal was observed in the group with mild dysfunction (−6.75%, *P* < 0.0004), moderate dysfunction (−13.17%, *P* < 0.0000), and severe dysfunction (−18.27%, *P* < 0.0223) according to the Di index. There were no significant differences in the fatigue of the right and left temporal muscles in each group of temporomandibular dysfunction according to the Di index (*P* < 0.0784).

Similar to the temporal muscles, changes in the mean power frequency of masseter muscles were the lowest in the group with no symptoms of dysfunction (−3.97%). Significantly higher muscle fatigue, as evidenced by a greater reduction in the mean power frequency, was found in groups with mild dysfunction (−13.15%, *P* < 0.0000), moderate dysfunction (−18.55%, *P* < 0.0000), and severe dysfunction (−23.02%, *P* < 0.0341) according to the Di index. As in the case of the temporal muscles, the impact of dysfunction on the differences in fatigue between the right and left masseter muscles has not been confirmed (*P* < 0.0937).

## 4. Discussion

Electromyography (EMG) is one of the few diagnostic tools that enable direct and objective assessments of muscle function. Practitioners dealing with functional disorders of the masticatory motor system are particularly interested in global electromyography (*surface electromyography* (SEMG)) because of the noninvasive nature of measurements that it provides [21–25].

Assessing susceptibility to muscle fatigue is a crucial element in the analysis of electromyographic examinations. Fatigue is usually defined as the point beyond which a particular level of force can no longer be maintained. Mean power frequency (MPF) and its changes linked to function are a reliable and objective indicator of muscle resistance to fatigue. Thus, changes in the frequency of the electrical activity of muscles, being a component of interference signal depletion, are a major predictor of susceptibility to muscle fatigue in EMG recordings. Muscle fatigue can also be determined by an increase in the EMG activity of muscles involved in generating a constant force. This is consistent with the view that generating a constant force as muscle fatigue increases must be associated with an increase in the electrical activity of muscles [26].

Our own examinations showed significant differences with regard to the type of muscles examined. There was a significantly greater depletion of the interference signal in respect of the changes in mean power frequency of the masseter muscles than the temporal muscles during 10 s of maximum isometric contraction in the intercuspal position.

Changes in the interference signal with respect to the mean power frequency of muscles during maximum isometric contraction were also a strong predictor of functional disorders in the masticatory motor system. Resistance to fatigue in the temporal and masseter muscles was significantly higher in the group with no symptoms of temporomandibular dysfunction than in the group of patients with symptoms of dysfunction according to the Di index. There was a significantly greater depletion of the interference signal for masseter and temporal muscles in the group with TMD.

Measurements of the mean power frequency of temporal and masseter muscles also showed high discriminatory efficiency for subjects with varying severities of temporomandibular dysfunctions according to the Di index. There were significant differences in terms of fatigue between the groups with varying severities of dysfunction for both temporal and masseter muscles.

The results of the study were based on the clinical index of temporomandibular dysfunction (Di). This index is simple and easy to use and is extensively used in research [27]. Although there are some limitations in using the Di, it represents a valid tool which correlates with the Research Diagnostic Criteria for Temporomandibular Disorders (RDC/TMD) in identifying patients with symptoms of TMD [16, 28].

In studies of masticatory muscle fatigue conducted by Sforza et al. [26] a muscle force sensor was used, which was located on one side between the dental arches of ten healthy subjects. This made it possible to terminate the effort test at the precise moment when the subjects could no longer produce the required bite force (127 N). As in previous studies, the endurance time ranging between 79 and 470 s in a group of ten examined subjects was a prognostic factor for muscle fatigue. An analysis of mean power frequency at the beginning and at the end of the test showed a significant decrease in the masseter muscles, which was not confirmed with regard to the temporal muscles. There was a significant decrease in the mean power frequency of both masticatory muscles after one minute on the side where the force sensor was placed.

Hori et al. [29] recorded mean power frequency (MPF) shift during fatigue and recovery of 46 healthy subjects and 46 patients with craniomandibular disorder at the beginning and the end of fatiguing clenching and then 3, 8, 13, and 18 min following the fatiguing clenching. The reference clenching force was 80% of each subject's maximal voluntary contraction (MVC). The results of the study showed significance between the healthy group and the group with craniomandibular disorder in the three following points, such as the mean of MPF values of the masseter muscles at the end of fatiguing clenching; the recovery pattern of the temporal muscles; and MPF shift induced by fatiguing clenching. These results therefore suggest that measuring fatigue and recovery MPF could be useful in the screening of craniomandibular disorders.

In studies conducted by Gay et al. [30] surface EMG recordings were made for both the masseter and anterior temporal muscles while the subject held an incisal bite force level of 10 N for as long as possible. The sample consisted of 18 patients with symptoms of TMD and 15 patients with no symptoms of TMD. The results showed that the endurance times were significantly shorter for the TMD patients; the masseter was not active in three of 17 TMD patients; and decreases in MPF over time were significantly greater for the TMD patients than normal subjects.

A study by Castroflorio et al. [31] concerned 20 healthy volunteers and 18 patients with TMD. An intraoral compressive-force sensor was used to measure the voluntary contraction forces close to the intercuspal position and to provide visual feedback of submaximal forces to the subject. Surface EMG signals were recorded with linear electrode arrays during isometric contractions at 20%, 40%, 60%, and 80% of the maximum voluntary contraction force, during an endurance test and during the recovery phase. The analysis of the results revealed that the temporal anterior and masseter muscle show the same myoelectric manifestations of fatigue and recovery and the initial values of the mean power frequency were lower in patients with muscle-related TMD.

Liu et al. [32] found significant differences in the mean power frequency of masseter and temporal muscles in a group of 24 subjects who had at least one objective or subjective symptom of masticatory system dysfunction compared to a group of 20 healthy people. Although an analysis of the results in both the examined groups showed a similar mean power frequency both at the beginning and at the end of maximum contraction in the intercuspal position over 30 s, there was a significantly greater decrease in the mean power frequency of the temporal muscles (right: 24.1 and left: 22.9) and masseter muscles (right: 19.2 and left 22.3) in the group with symptoms of TMD in comparison to the group with no symptoms of TMD (temporal muscles: right 13.4 and left 15.3; masseter muscles: right 10.5 and left 10.9).

The studies presented, whose observations are consistent with the results of our own findings, provide justification for using the analysis of muscle fatigue in the identification and discrimination of subjects with symptoms of masticatory system dysfunction.

## 5. Conclusions

1. The results of the presented study showed an increase in the fatigue of temporal and masseter muscles in direct proportion to the severity of symptoms of temporomandibular dysfunction in the examined patients.
2. The use of surface electromyography in the assessment of muscle fatigue is an excellent diagnostic tool for identifying patients with temporomandibular dysfunction.

## Figures and Tables

**Table 1:** The exclusion criteria adopted in anamnesis and the number of patients included in the study.

Total number of patients participated in the study		200

Exclusion criteria	Depressive disorders	0
	Pain in other parts of the body	4
	Inflammations	3
	Taking painkillers and antidepressants	1
	Periodontal diseases	1
	Completed treatment of masticatory motor system dysfunctions	2
	Completed orthodontic treatment	15

Total number of patients included in the study		174

**Table 2:** Clinical index of temporomandibular dysfunction (Di).

Di	Symptoms
Mandibular movements	
0	Normal range
1	Small reduction in amplitude
5	Large reduction in amplitude

Temporomandibular joint function	
0	Smooth, noiseless abduction and adduction of mandible, trajectory asymmetry <2 mm
1	Noise in one joint or both joints during abduction and adduction of mandible, trajectory asymmetry >2 mm
5	Abduction of mandible impossible and/or luxation

Masticatory muscle pain	
0	No tenderness
1	Tenderness of 1–3 sites
5	Tenderness of 4 and more sites

Temporomandibular joint pain	
0	No tenderness
1	Unilateral or bilateral tenderness
5	Unilateral or bilateral tenderness of the dorsal surface of joint

Pain during movement of mandible	
0	No pain
1	Pain during one out of all possible movement directions
5	Pain during more than one out of all possible movement directions

**Table 3:** Interpretation of the clinical index of temporomandibular dysfunction (Di).

Range	Severity of dysfunction	Description
0	Di 0	No dysfunction
1–4	Di I	Mild dysfunction
5–9	Di II	Moderate dysfunction
10–25	Di III	Severe dysfunction

**Table 4:** Changes in mean power frequency (MPF%) of muscles during 10 s of maximal voluntary contraction in intercuspal position depending on the temporomandibular dysfunction index Di.

		Di group											
Side/gender		0			I			II			III		
		*n*	Mean	SD	*n*	Mean	SD	*n*	Mean	SD	*n*	Mean	SD
Temporal muscles													
Left	Females	22	−2.18	6.99	39	−6.64	5.13	25	−14.71	5.81	7	−10.44	9.14
	Males	23	−2.80	3.54	29	−6.10	5.34	23	−11.49	6.17	6	−27.83	0.82
	Total	**45**	**−2.50**	**5.45**	**68**	**−6.41**	**5.19**	**48**	**−13.17**	**6.14**	**13**	**−18.47**	**11.11**
Right	Females	22	−3.55	7.82	39	−7.39	6.11	25	−15.03	4.04	7	−11.70	3.56
	Males	23	−3.10	4.29	29	−6.66	5.64	23	−11.14	4.87	6	−25.48	1.67
	Total	**45**	**−3.34**	**6.20**	**68**	**−7.08**	**5.88**	**48**	**−13.17**	**4.83**	**13**	**−18.06**	**7.66**

Masseter muscles													
Left	Females	22	−1.89	7.51	39	−12.99	7.95	25	−19.51	4.81	7	−9.79	10.23
	Males	23	−5.30	4.67	29	−10.95	4.14	23	−17.80	3.72	6	−36.77	7.43
	Total	**45**	**−3.64**	**6.39**	**68**	**−12.12**	**6.63**	**48**	**−18.69**	**4.36**	**13**	**−22.24**	**16.47**
Right	Females	22	−3.35	10.25	39	−14.85	8.80	25	−19.09	4.51	7	−14.77	4.18
	Males	23	−5.20	5.90	29	−13.31	4.77	23	−17.66	3.37	6	−34.32	7.31
	Total	**45**	**−4.30**	**8.27**	**68**	**−14.19**	**7.35**	**48**	**−18.40**	**4.03**	**13**	**−23.79**	**11.57**
